# Highly Responsive Pd-Decorated MoO_3_ Nanowall H_2_ Gas Sensors Obtained from In-Situ-Controlled Thermal Oxidation of Sputtered MoS_2_ Films

**DOI:** 10.1021/acsami.2c04804

**Published:** 2022-05-24

**Authors:** Soheil Mobtakeri, Saman Habashyani, Emre Gür

**Affiliations:** † Department of Nanoscience and Nanoengineering, Graduate School of Natural and Applied Science, Atatürk University, Erzurum 25240, Turkey; ‡ Department of Physics, Faculty of Science, Ataturk University, Erzurum 25250, Turkey

**Keywords:** thermal oxidation, RF-magnetron sputtering, MoO_3_, MoS_2_, H_2_ gas sensor, gasochromism

## Abstract

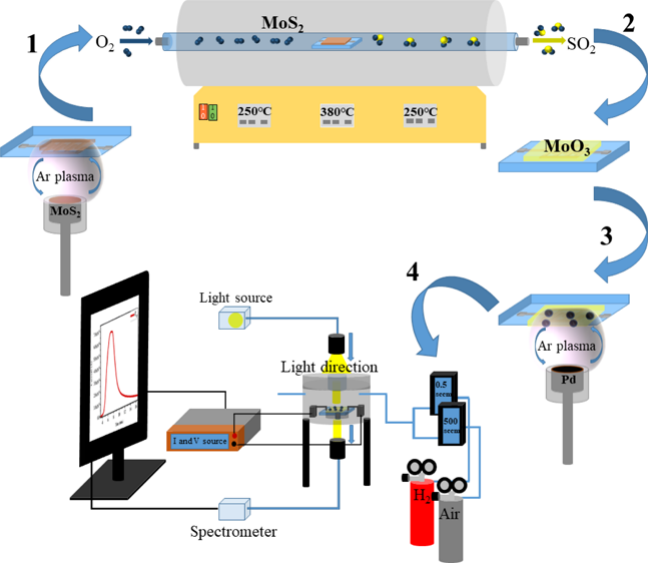

Among transition metal oxides, MoO_3_ is a promising material due to its layered structure and different oxidation states, making it suitable for different device applications. One of the methods used to grow MoO_3_ is radio frequency magnetron sputtering (RFMS), which is the most compatible method in industry. However, obtaining nanostructures by RFMS for metal oxides is challenging because of compact morphology film formation. In this study, α-MoO_3_ with vertical nanowalls is obtained by a two-step process; deposition of magnetron-sputtered MoS_2_ vertical nanowalls and postoxidation of these structures without changing the morphology. In situ transmittance and electrical measurements are performed to control the oxidation process, which shed light on understanding the oxidation of MoS_2_ nanowalls. The transition from MoS_2_ to α-MoO_3_ is investigated with partially oxidized MoS_2_/MoO_3_ samples with different thicknesses. It is also concluded that oxidation starts from nanowalls perpendicular to the substrate and lasts with oxidation of basal planes. Four different thicknesses of α-MoO_3_ nanowall samples are fabricated for H_2_ gas sensors. Also, the effect of Pd deposition on the H_2_-sensing properties of sensors is deeply investigated. An outstanding response of 3.3 × 10^5^ as well as the response and recovery times of 379 and 304 s, respectively, are achieved from the thinnest Pd-loaded sample. Also, the gas-sensing mechanism is explored by gasochromic measurements to investigate the sensor behaviors under the conditions of dry air and N_2_ gas as the carrier gas.

## 1 Introduction

Transition metal oxides such as WO_3_, MoO_3_, and V_2_O_5_ are functional materials that has been attracting the attention of scientists in the last few years due to their wide and tunable band gaps, variable electronic and optical properties due to reduction and oxidation properties, as well as their layered structure in hydrate or anhydrate crystal phases.^1^ Among them, MoO_3_ and its substoichiometric forms have a wide range of applications such as in electrochromic devices,^2−4^ gas sensors,^5−7^ optoelectronics,^8^ catalysis,^9^ supercapacitors,^10^ and solid lubricants.^11^ Stoichiometric MoO_3_ is a wide band gap semiconductor (∼3 eV) that might be narrowed by reduction into MoO_*x*_ (2 < *x*< 3).^12,13^ In the reduction process, oxygen defects introduce more electrons into the conduction band. This presents itself as the narrowed band gap and better conductivity, which become possible by Mo^6+^ reduction to Mo^5+^ and finally to Mo^4+^ to achieve semimetal MoO_2_.^13^ Through this process, the optical properties of MoO_3_ change significantly. Therefore, it is among the most studied materials for various devices, especially electrochromic and gasochromic devices.

MoO_3_ has two thermodynamically stable common crystal phases, stable orthorhombic α-MoO_3_ and metastable monoclinic β-MoO_3_.^12^ The α-phase attracts much attention due to its layered crystal structure and its ability to form two-dimensional (2D) atomically thin morphologies, which is a state-of-the-art material after discovering graphene.^13^ The fundamental building blocks of both α-MoO_3_ and β-MoO_3_ crystal structures are MoO_6_ octahedrons that bond differently.^14^ In orthorhombic α-MoO_3_, distorted MoO_6_ octahedrons link with edge-sharing zigzag rows and are corner-sharing along [001] and [010] directions, respectively, to form planar double-layer sheets by weak van der Waals force between them.^15,16^ Unlike the α-phase, monoclinic β-MoO_3_ does not form a layered crystal structure, and MoO_6_ octahedrons only share corners to form a ReO_3_-like crystal structure by corner-sharing.^13,15^ Depending on the applications, α-MoO_3_ and β-MoO_3_ have their own advantages and disadvantages. The transition from the β-phase to the α-phase at temperatures above 350 °C has been reported.^17^

Understanding the shape and size effects of materials on their properties and tracing their behaviors in contact with the environment around them have enabled scientists fabricate different nanostructures with improved optoelectronic properties, which led to the fabrication of materials with a high aspect ratio. In the case of MoO_3_, nanostructures such as nanosheets,^18^ nanorods,^19^ nanowires,^20^ quantum dots,^21^ nanotubes,^22^ and nanoparticles^23^ have been reported so far via different material growth methods such as chemical vapor deposition,^24^ radio frequency (RF)/DC magnetron sputtering,^25^ thermal evaporation,^26^ electron beam evaporation,^27^ spray pyrolysis,^28^ hydrothermal synthesis,^29^ electrochemical anodization,^30^ electrochemical deposition,^15^ and exfoliation.^31^ All the material growth methods mentioned above have their advantages and disadvantages. However, the sputtering method has some advantages that distinguish it from the growth methods mentioned above, such as its compatibility with industry, large area coatings, and the possibility of mass production. In addition, it is easy to control growth parameters to perform uniform and repeatable depositions. On the other hand, it is also inexpensive compared to other high vacuum-required growth methods. Besides these advantages, it is challenging to grow nanostructures by tuning the controllable growth parameters in magnetron sputtering. It is common that sputtered materials form a compact thin film morphology. In order to achieve nanostructures via the magnetron sputtering method, some extra post-treatments, usage of catalysis, and substrate treatments might be required. For example, Zhang et al. prepared monolayer hollow spherical tungsten oxide films by combining magnetron sputtering with the colloidal crystal template.^32^ Instead of growing MoO_3_ nanostructures by the methods mentioned above, there are also some reports on the full or partial oxidation of MoS_2_ to achieve MoO_3_ or MoS_2_/MoO_3_ hybrid composite structures. In one of them, 2D MoO_3_ nanosheets were synthesized by chemical oxidation of exfoliated MoS_2_.^3^ Similarly, MoS_2_/MoO_3_ heterostructures were prepared by plasma oxidation of MoS_2_ layers.^33^ On the other hand, the thermal oxidation method is preferred by some researchers.^34−37^ However, most of them did not successfully preserve the morphology during the conversion from MoS_2_ to MoO_3_ material.^38,39^

One of the most used applications of MoO_3_ materials is the gas sensor devices.^40−42^ Metal oxide semiconductors are preferred because of their resistance to a harsh environment, ease of production, low cost, and repeatability. Also, due to the ease of production and the ability to miniaturize, chemiresistive semiconducting metal oxide gas sensors and their heterostructures attract much attention among other types of gas sensors.^43^ In particular, materials for H_2_ gas sensing have gained importance because they are supposed to be one of the materials with the highest potential as alternative energy sources. Increasing the usage of H_2_ gas as an alternative to fossil fuels and considering its explosive potential, detecting H_2_ gas leakages in storage containers and gas tanks at low concentrations (under 1000 ppm) and relatively low temperatures becomes highly important. H_2_ is an odorless gas, which has a high propagation constant, a low ignition energy, and a wide burning range (4–75%) in air.^44^ A variety of metal oxides such as SnO_2_,^45^ WO_3_,^46,47^ MoO_3_,^18,48,49^ ZnO,^50^ and In_2_O_3_^51^ have been utilized for H_2_ gas sensors. Because of the safety concern of H_2_ gas, it is very important to achieve high responses against low concentrations at low working temperatures of H_2_ gas. It is important to achieve easily readable current values in the case of chemiresistive gas sensors. The oxide-based sensors generally operate at relatively high temperatures with low sensitivity.^52^ Pd-decorated MoO_3_ has become a promising material among the metal oxide semiconductor gas sensors for detecting H_2_ gas due to the chromic nature of MoO_3_ and its ability to undergo reversible reduction and oxidation reactions by ion injection and extraction.

Here, we report thermal oxidation of sputtered MoS_2_ to achieve α-MoO_3_ without a morphological change of the film to use in highly responsive H_2_ gas sensors. In situ conversion of MoS_2_ to MoO_3_ was performed through optical and electrical measurements. Furthermore, the potential of the converted materials was explored by fabricating H_2_ gas sensors with different thicknesses to study the effect of thickness and nanostructures on the gas-sensing properties of achieved MoO_3_. An outstanding sensitivity of 3.3 × 10^5^ for 1000 ppm was achieved from the thinnest MoO_3_ sample, one of the highest reported responses for MoO_3_-based H_2_ gas sensors at a relatively low operating temperature (100 °C). Furthermore, the effect of Pd deposition on H_2_ gas-sensing performance is discussed in depth in the H_2_ Gas-Sensing Performances and Mechanism section.

## 2 Experimental Section

The Experimental Section in this study is divided into four parts: (a) film deposition, (b) dynamic in situ transmittance and electrical measurements during the oxidation process, (c) film characterization, and (d) H_2_ gas sensor measurements.

### 2.1 Film Deposition

MoS_2_ nanowalls were deposited via a RF magnetron sputtering (RFMS) system using a MoS_2_ target with a diameter of 2 in. and a purity of 99.99%. Quartz and ITO-coated glasses with a sheet resistance of 20 Ohm/cm were used as substrates for gas sensor measurements and material characterizations. Both quartz and ITO substrates were cleaned ultrasonically in isopropanol, ethanol, and deionized water (10 min for each step) and dried under N_2_. The distance between the target and substrates was about 7 cm, and the gun position was fixed perpendicular to the substrates. The chamber was evacuated to a base pressure of 2 × 10^–6^ Torr. Sputtering was performed at a substrate temperature of 300 °C and an argon pressure of 16 mTorr with a sputtering power of 120 W. Four different thicknesses of MoS_2_ were sputtered. Depending on the growth time, films were labeled Z_0.5_, Z_2.5_, Z_7.5_, and Z_30_, corresponding to 30 s, 2.5 min, 7.5 min, and 30 min growth time with thicknesses of 40, 115, 370, and 1440 nm, respectively, as shown in the cross-sectional field emission scanning electron microscopy (FESEM) images presented in Supporting Information, Figure S1.

### 2.2 In Situ Transmittance and Electrical Measurements

The dynamic in situ transmittance and two-probe electrical measurements were performed during the oxidation process in a chamber having an optical window. A tungsten–halogen lamp was used as the light source. MoS_2_ samples to be oxidized were heated up to 380 °C, and oxygen gas was let in during preheating up to 380 °C and during the oxidation process. For two-probe electrical measurements, interdigitated electrode (IDE) contacts (Pt contacts deposited with a RF-magnetron sputter) with a 400 μm distance between them were used.

### 2.3 Film Characterization

A Zeiss Sigma 300 field emission scanning electron microscope was used to investigate the morphological characteristics of MoS_2_ and MoO_3_ films. A WITech alpha 300R was used for micro-Raman measurements of MoS_2_, MoS_2_/MoO_3_, and MoO_3_ films. For crystal structure characterization of films, a PANalytical Empyrean X-ray diffraction (XRD) system was used. X-ray photoelectron spectroscopy (XPS) measurements for determining the material composition were performed with a Specs Flex-Mod XPS system equipped with a 150 mm radius hemispherical energy analyzer with a 2D charge-coupled detector. The measurements were performed using an Al anode with a K_α_ energy of 1486.71 eV. The depth profile measurements were conducted by 3 keV Ar^+^ ion sputtering with 300 s of etching for each cycle.

### 2.4 H_2_ Gas Sensors (Sample Preparation and Performance Measurements)

For H_2_ gas sensor measurements, Pt IDE contacts were deposited first on a quartz substrate by sputtering using a shadow mask. Then, MoS_2_ films with different thicknesses were deposited on contacts. Next, oxidation of samples of different thicknesses was performed in tube furnaces for 2 h. At the final step of the device fabrication, sample surfaces were activated by Pd deposition under 27 mTorr and 25 W power conditions for 7 s by RFMS. Gas sensor measurements were performed using a homemade gas sensor system with a chamber of 0.5 L volume. All the samples were held under an applied voltage of 0.5 V using a KEITHLEY 487 picoammeter/voltage source. H_2_ gas with various standard cubic centimeters per minute (sccm) was mixed with 500 sccm of dry air to achieve the desired H_2_ part per million (ppm) levels between 100 and 1000 ppm at three different measurement temperatures (100, 200, and 300 °C), which were set before the gas-sensing measurements. Measurements between 10 and 90 ppm were performed for sample Z_0.5_ at an operating temperature of 200 °C.

## 3 Results and Discussion

### 3.1 In Situ Measurements

The thermal oxidation process was used to convert sputtered nanowall morphology MoS_2_ films into MoO_3_ without changing the surface morphology to take advantage of the large surface-to-volume ratio of the films in sensor applications. After many trials to keep the surface morphology with no change (Supporting Information, Figure S2), it has been realized that certain oxidation conditions are needed to control during the thermal oxidation, such as the oxidation temperature and time. It has also been confirmed that these parameters depend on the MoS_2_ thickness. Therefore, it is decided to control the oxidation process by in situ transmittance and two-probe electrical measurements. These in situ measurements allow us to trace the sulfur-to-oxygen ratio by simply checking the samples’ opaqueness because the decreasing sulfur to oxygen ratio presents itself as increasing transparency and decreasing conductivity. The predictable chemical equation for the thermal oxidation process of MoS_2_ is shown below
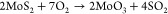


In some applications, it is inevitable to use transparent conductive oxides (TCOs) as a substrate for MoO_3_ films, that is, electrochromic devices, in which the transparency and conductivity of electrodes are essential parameters. Moreover, a longer oxidation time means the disappearance of oxygen vacancies in TCO, which presents itself as a decrease in conductivity.^53,54^ Therefore, for such situations, controlling the oxidation time might be an important parameter.

**Figure 1:**
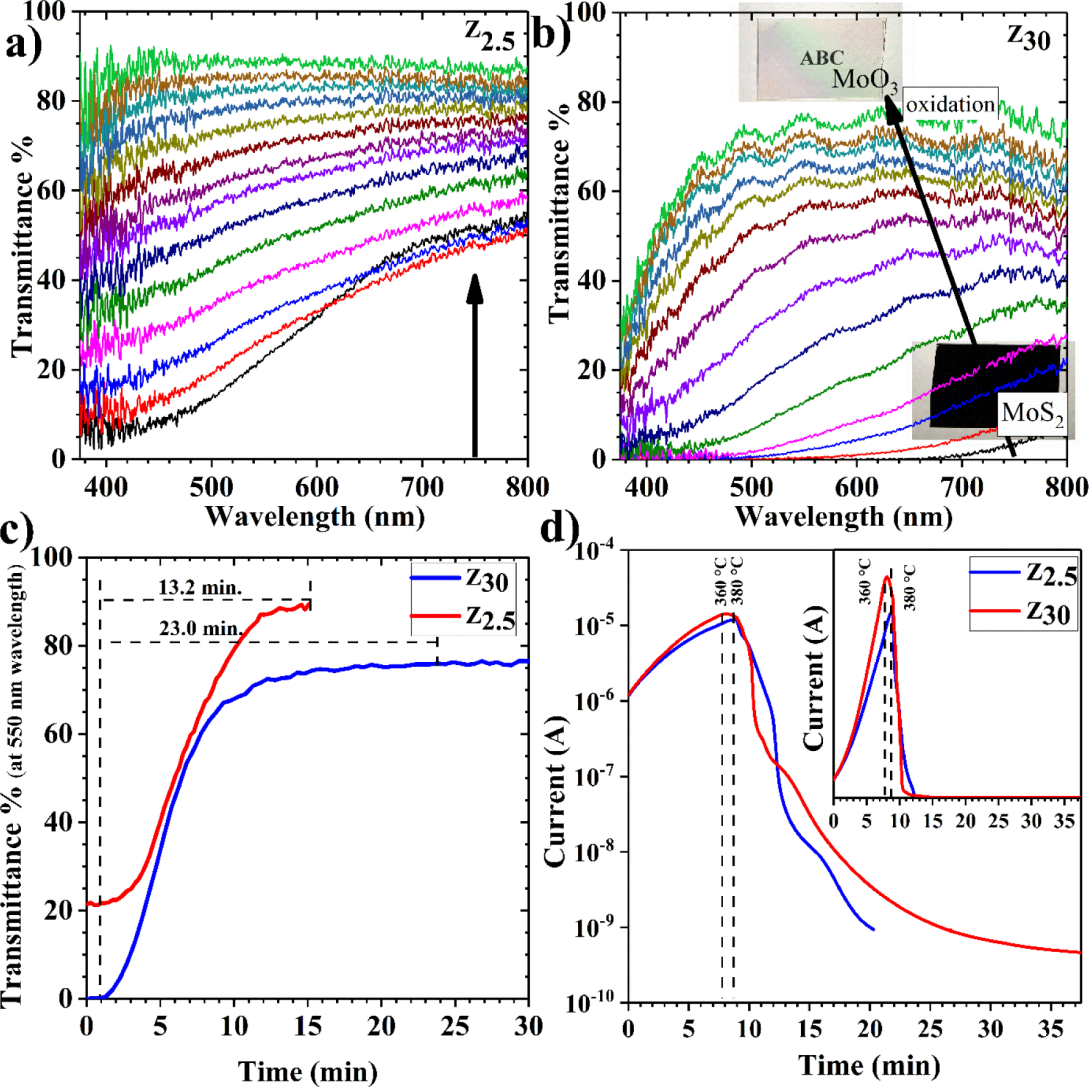
In situ optical and electrical measurements during oxidation. (a) Transmittance change for sample Z_2.5_, (b) transmittance change for sample Z_30_, (c) transmittance changes of Z_2.5_ and Z_30_ vs time at a wavelength of 550 nm, and (d) current changes of Z_2.5_ and Z_30_ during the oxidation vs time.

MoS_2_ films with two different thicknesses, namely, Z_2.5_ and Z_30_ were used for in situ measurements. Changes in optical and electrical properties of MoS_2_ films with the transmittance during the oxidation process are shown in Figure 1a,b. The data collection time for the transmittance change is every 60 s. In order to avoid crowd plots, sparse data is used in the plot. As seen in Figure 1a,b, there is a significant change in the transparency of the films, which turned from opaque to transparent (see the inset image). This indicates that the oxidation process is successful. Figure 1c shows the transmittance change of films versus time monitored at a wavelength of 550 nm. As seen from the figures, both samples’ transparency increase as the oxidation time gets longer. The films’ starting transmittance values change due to the thickness differences in the samples. This is so crucial for the research that we have conducted because it allows us to find approximate oxidation time. The term approximation is used here because finding the exact oxidation time from the graph is hard. The change in transmittance becomes slower after some duration, as seen from the plot. At this point, it is not easy to understand whether all sulfurs are gone or some remain. On the other hand, this plot, in its way, gives a strong idea about the approximate oxidation time. As seen from Figure 1c, the oxidation times for Z_2.5_ and Z_30_ are about 13.2 and 23.0 min, respectively. The differences in oxidation times between Z_2.5_ and Z_30_ are predictable due to the thickness differences between the films. It can be seen that the slope of Z_30_ is greater than that of Z_2.5_, which means faster oxidation. In addition, the tendency of dynamic transparency of Z_30_ gives rise to flattening quicker than that of the sample Z_2.5_, but transmittance of Z_30_ still increases very slowly.

Figure 1d shows the current versus time changes of MoS_2_ films during the oxidation process. It can be noted that the graph is semilogarithmic. As seen from the plot, the current value decreases as the oxidation time gets longer, which shows basically that converted MoO_3_ films have a larger resistance than MoS_2_. As seen from the figure for sample Z_2.5_, current increases from 1.2 to 12.0 μA. The increase in the current value in this interval is related to the increase in the temperature from RT to 380 °C. However, the temperature reached at the maximum point of the current is 375 °C. The current starts to decrease rapidly at a temperature of 375 °C, indicating that the oxidation process has started handling the current decrease. After 586 s, the current value reaches 1.7 nA for the sample Z_2.5_. For sample Z_30_, the current starts from 1.2 μA and reaches a value of 14.2 μA. Unlike the sample Z_2.5_, the current starts to decrease for Z_30_ at a temperature of 360 °C. The Z_30_ sample takes about 890 s to achieve a current value of 1.7 nA. This experiment proves a successful oxidation process with the in situ measurements and gives some information about the conductivity of the samples. It also allows us to stop the oxidation process by checking the sample’s transparency or conductivity to obtain a semioxide composite of MoS_2_/MoO_3_.

### 3.2 Material Characterization

**Figure 2:**
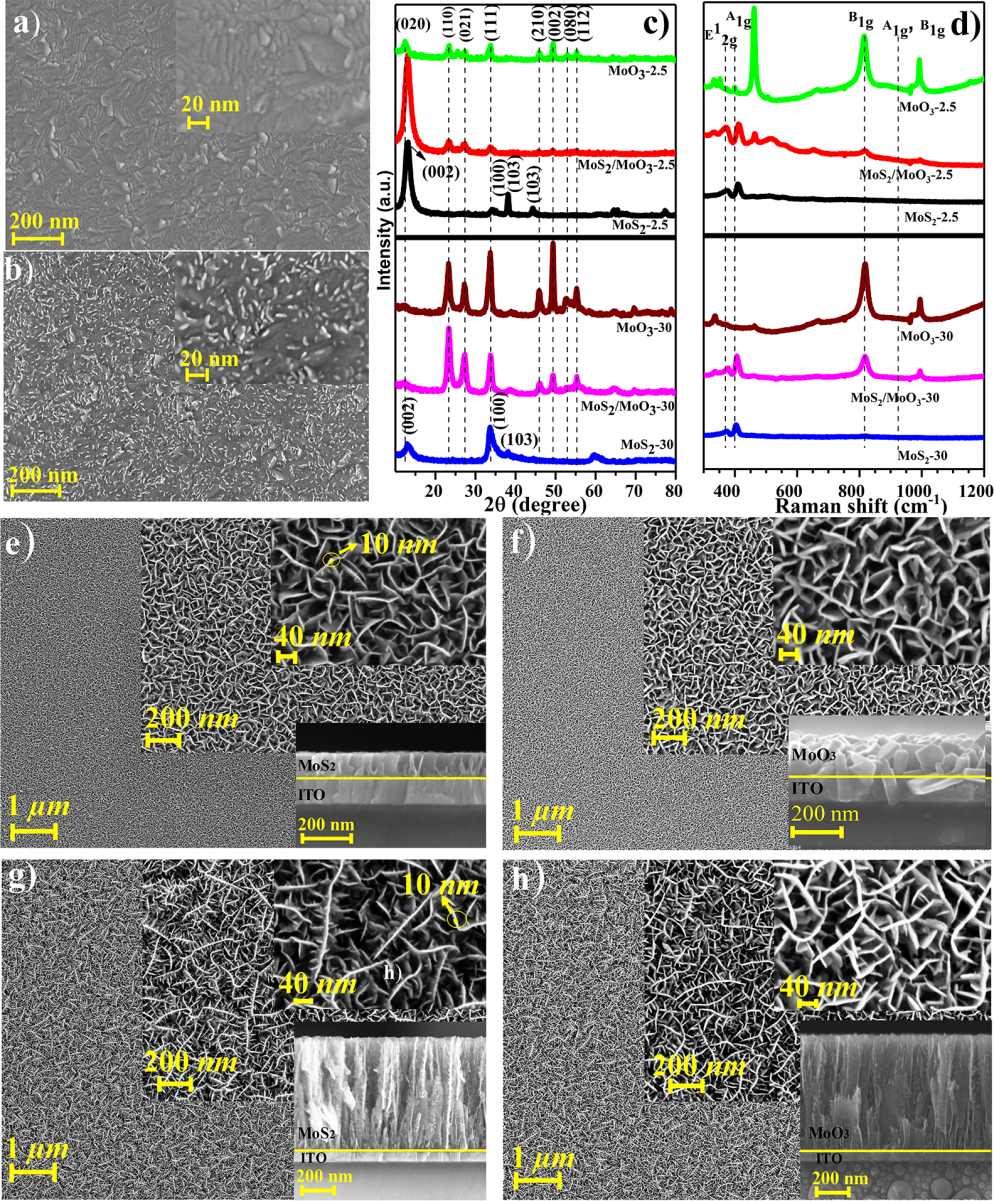
(a,b) FESEM images of 15 and 30 s MoS_2_ grown on the ITO film, respectively. (c,d) XRD and Raman shifts of MoS_2_, MoS_2_/MoO_3_, and MoO_3_ for 2.5 and 30 min grown films, respectively. (e,f) FESEM and cross-sectional FESEM images of 2.5 min grown MoS_2_ and MoO_3_ after thermal oxidation, respectively. (g,h) FESEM and cross-sectional FESEM images of 30 min grown MoS_2_ and MoO_3_ after thermal oxidation, respectively.

In situ optical and electrical measurements confirm the successful thermal oxidation process of MoS_2_ into MoO_3_. In order to follow the morphology, as well as the structural and optical properties after the MoS_2_–MoO_3_ conversion, Raman, XRD, and the FESEM measurements were performed on the end materials together with the MoS_2_/MoO_3_ composite materials in which the thermal oxidation process was completed at some point. Figure 2a–h shows the results of these measurements. However, before we elaborate on how oxidation takes place in MoS_2_ nanowalls, first, we offer a glimpse on the growth mechanism of MoS_2_ nanowalls. Figure 2a,b presents FESEM images of 15 and 30 s grown MoS_2_ films on the ITO substrate. As seen from Figure 2a, no vertically grown MoS_2_ nanowall-shaped morphology is seen in 15 s of deposition, but ITO film grains can be seen. The result is because of horizontally grown MoS_2_ with a 2D-like morphology at the first step of deposition. As the growth time continues, the nanowall-like vertical structures start to appear, as seen in 30 s grown MoS_2_. The appearance of 3D structures might be due to accruing stress between boundaries of grown sheets or defect sites that start during the first steps of deposition. After a while, it appears to be the beginning of vertical growth.^55^ A detailed study of transition from 2D to 3D growth forming nanowalls in the WS_2_ film deposited by RFMS, which was predicted to be the same for MoS_2_, was reported in our previous study.^55^

Figure 2c shows the XRD graphs of MoS_2_, partially oxidized MoS_2_/MoO_3_, and MoO_3_ films of Z_2.5_ and Z_30_ samples. The (002) plane peak intensity, related to basal planes oriented parallel to the substrate, has decreased with a longer deposition time. However, the peak intensity of the (100) plane, which is related to edge planes perpendicular to substrates, increases as the deposition time gets longer.^56^ This might also prove the 2D and 3D growth stages in sputtered MoS_2_. A similar observation of 2D and 3D growth stages has been reported earlier.^57^ This structural anisotropy in MoS_2_ films also gives rise to anisotropic oxidation. It is known that oxidation of MoS_2_ starts from edge crystal planes, and oxidation is very easy for these planes compared to the basal planes.^58^ The (002) basal plane is more protected against oxidation. However, as already reported, the edge plane, that is (100), is in more contact with the environment and is more reactive to oxidation.^59^ As shown in Figure 1c, there is no linear change in the oxidation time with increasing thickness. The oxidation time for Z_2.5_ is 13.2 min, while it is 23.0 min for Z_30_.

This result is because of the formation of the same (002) basal plane in both thin and thick films, where the first nanowalls are extremely fast oxidized, followed by very slow oxidation of the (002) basal plane. As shown in Figure 2e,g, the thickness of walls is approximately about 10 nm making oxidation easier. In addition, sputter-grown MoS_2_ films are highly sulfur-deficient,^60^ and this might be helpful for the oxidation process. XRD patterns show that the edge plane (100) is almost diminished in the partially oxidized MoS_2_/MoO_3_ sample. However, the (002) plane can still be observed, belonging to the MoS_2_ basal plane, which indicates that the oxidation starts from the edge planes. Furthermore, it can be seen that MoO_3_-related XRD peaks start to appear. Overall, all the MoS_2_-related XRD peaks entirely disappear, and the appearance of the (020) peak and several other XRD peaks belonging to the oxide indicates the successful transformation into α-MoO_3_.

Figure 2d shows the Raman spectra of MoS_2_, MoS_2_/MoO_3_ composite structure, and MoO_3_. Both E_2g_^1^ and A_1g_, which are related to in-plane and out-of-plane Raman optical phonon modes of MoS_2_, respectively, appear for each different thickness. B_1g_ and A_1g_ modes of α-MoO_3_ start to appear for the partially oxidized MoS_2_/MoO_3_ structure. After oxidation, all the MoS_2_-related Raman peaks disappear, indicating that fully oxidized α-MoO_3_ is achieved. The primary purpose of this study is to investigate the oxidation of MoS_2_ nanowalls without changing the morphology of films. Figure 2e–h shows the FESEM images of 2.5 and 30 min grown MoS_2_ films and MoO_3_ after oxidation. The FESEM images of MoS_2_ show the homogeneous distribution of nanowalls throughout the substrate. It is also seen from FESEM images that increasing the deposition time gives rise to extra branches growing on nanowalls. Cross-sectional SEM images also show vertically grown nanowalls (for cross-sectional FESEM images of Z_2.5_, Z_7.5_, Z_10_, and Z_30_, see Supporting Information, Figure S1). From transmission electron microscopy images of obtained MoO_3_, it can be seen that the Mo to Mo distance between two layers is about 1.37 nm (Supporting Information, Figure S3) which is close to the theoretical value of 1.4 nm.^13^Figure 2f,h shows the FESEM images of thermally oxidized MoO_3_ grown for 2.5 and 30 min. As seen from FESEM images, there is no significant change in the morphology of oxide films compared to the sulfide films.

**Figure 3:**
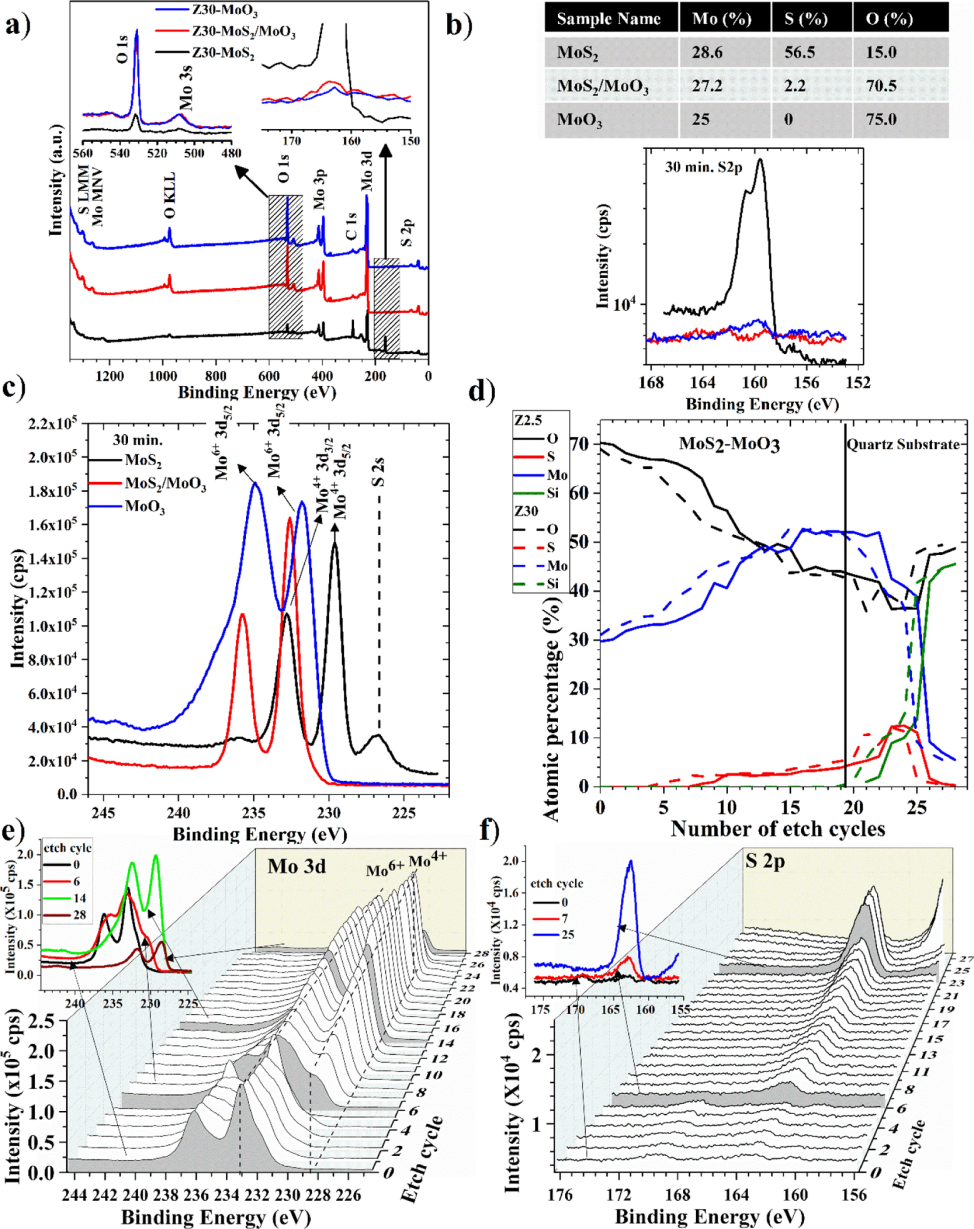
30 min grown MoS_2_, partially oxidized MoS_2_/MoO_3_, and fully oxidized α-MoO_3_ samples. (a) XPS survey spectra, (b) high-resolution spectra of the S element, and (c) high-resolution spectra of the Mo element. Depth profile for the 30 min grown and partially oxidized MoS_2_/MoO_3_ sample, (d) atomic percentage variation with the depth profile for Mo, S, and O elements, (e) Mo 3d region, and (f) S element 2p region.

Figure 3a shows the XPS survey spectra of 30 min grown MoS_2_, fully oxidized α-MoO_3_, as well as the partially oxidized MoS_2_/MoO_3_ sample. The figure shows only peaks belonging to the C, O, S, and Mo elements. The C element peak is attributed to surface contamination. This adventitious C–C binding energy is used to correct the spectra obtained. The inset figure shows the energy regions of O and S elements in the survey spectra. The intensity of the O element peak is the weakest in the MoS_2_ sample, which is due to the surface oxidation, and it is increased for the oxidized samples. On the other hand, the S element peak is the maximum for the MoS_2_ sample, as shown in the inset and the high-resolution spectra in Figure 3b, while it diminishes for the fully oxidized α-MoO_3_ sample. The partially oxidized MoS_2_/MoO_3_ sample shows minimal S element peak intensity as seen in the spectra, although we tried to obtain a more significant intensity by taking out the sample from oxidation in a few minutes. Nevertheless, it is realized that the surface is fully oxidized upon starting the oxidation because the nanowall morphology of MoS_2_ is highly active for oxidation, which is already discussed in the XRD analysis. The calculated atomic percentages of the elements are given in the inset table. This data clearly shows the successful oxidation of the sputtered MoS_2_ samples. Figure 3c shows the high-resolution Mo 3d region spectra of the three samples investigated in the study. An S 2s peak is seen for the MoS_2_ sample, while no signal appears for the fully and partially oxidized samples. It is apparent in the figure that the Mo binding energy peak appears on the lower energy side, indicating the Mo^4+^ oxidation state of the MoS_2_ sample. This confirms the formation of MoS_2_. On the other hand, partially and fully oxidized samples show similar binding energy peaks, shifting to a higher binding energy corresponding to the Mo^6+^ oxidation state. This confirms the formation of MoO_3_ after thermal oxidation.

Figure 3d–f shows the depth profile of the 30 min partially oxidized sample, which shows how the oxidation process occurs. The Mo 3d high-resolution depth profile is obtained for a total of 30 cycles of measurements. The Mo 3d binding energy peaks appear at 233.1 and 236.1 eV, which are similar up to the sixth etch cycle. These binding energy values correspond to the Mo^6+^ oxidation state, confirming the formation of MoO_3_.^61^ As seen in Figure 3e, the Mo peak displays a binding energy shift as the etch cycle number increases. At a lower binding energy, the shoulder starts to appear at the sixth etch cycle. The evolution of the shoulder peak can be clearly seen in the spectra in which it first appears as a shoulder, then becomes more prominent in terms of intensity, and finally becomes a separate dominant peak. Although some reports show the reduction of MoO_3_ to lower oxidation states with Ar^+^ ion sputtering, it can be seen in the S element depth profile in Figure 3f that the S element starts to appear at the sixth etch cycle, where exactly a shoulder peak appears first in the Mo 3d binding energy region. The inset in Figure 3e shows the Mo 3d spectra for some etch cycles. A shift of the binding energy peak for the entire depth of the sample to a lower binding energy region corresponding to the lower oxidation states of the Mo element can be seen in this inset figure clearly. In the S element’s depth profile, the S element appears at the sixth cycle and becomes highly dominant as the number of etch cycles is increased. These two Mo and S elements’ depth profiles indicate that oxidation of MoS_2_ starts from the surface and continues for the entire depth of the sample, as previously discussed in the XRD data. It is clear that oxidation did not definitely occur beyond the 14th etch cycle for the partially oxidized MoS_2_/MoO_3_; beyond that cycle, bare, not oxidized MoS_2_ is present, which is confirmed by the Mo 3d and S 2p photoelectron binding energy peaks.

### 3.3 H_2_ Gas-Sensing Performances and Mechanism

**Figure 4:**
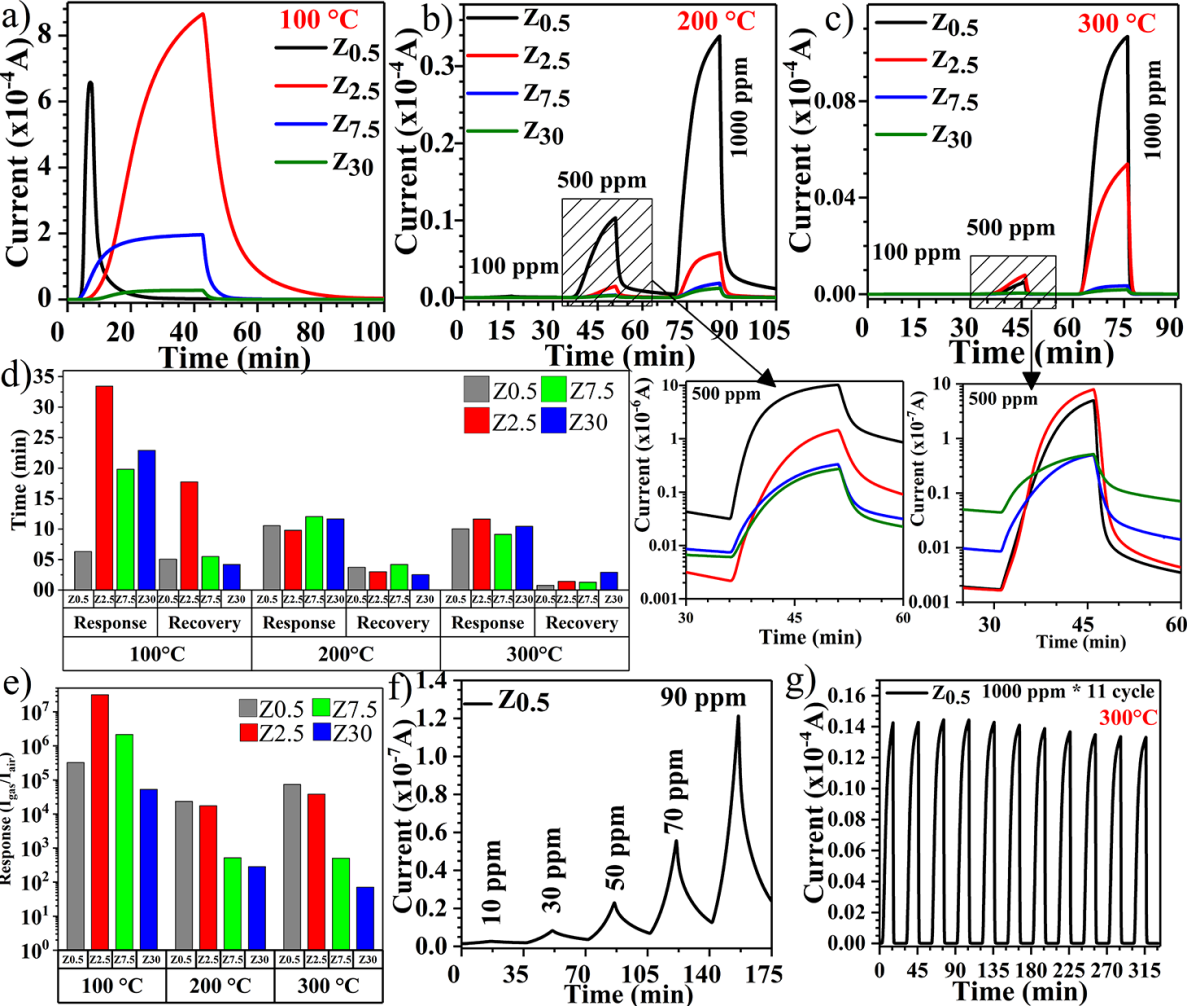
(a) Dynamic responses of Z_0.5_, Z_2.5_, Z_7.5_, and Z_30_ for 1000 ppm H_2_ gas at an operating temperature of 100 °C. (b) Dynamic responses of Z_0.5_, Z_2.5_, Z_7.5_, and Z_30_ for 100, 500, and 1000 ppm H_2_ gas at an operating temperature of 200 °C. (c) Dynamic responses of Z_0.5_, Z_2.5_, Z_7.5_, and Z_30_ for 100, 500, and 1000 ppm H_2_ gas at an operating temperature of 300 °C. Insets show 200 and 300 °C semilogarithmic dynamic responses for the 500 ppm H_2_ level. (d) Response and recovery times of Z_0.5_, Z_2.5_, Z_7.5_, and Z_30_ for 1000 ppm H_2_ concentration at different operating temperatures. (e) Response bar of Z_0.5_, Z_2.5_, Z_7.5_, and Z_30_ for 1000 ppm H_2_ concentration at different operating temperatures. (f) Dynamic responses of Z_0.5_ at an operating temperature of 200 °C for H_2_ concentrations under 100 ppm. (g) 11 Cycles for Z_0.5_ at an operating temperature of 300 °C for 1000 ppm H_2_ concentration.

MoO_3_ films with four different thicknesses corresponding to their deposition times of 0.5, 2.5, 7.5, and 30 min, indicated as Z_0.5_, Z_2.5_, Z_7.5_, and Z_30_, respectively, are studied. Figure 4a–e shows dynamic responses, response, and recovery times, and response (*I*_H_2_gas_/*I*_air_) of MoO_3_ sensors at different operating temperatures and for different H_2_ gas concentrations. Figure 4a shows dynamic responses of MoO_3_ nanowalls different thicknesses for 1000 ppm H_2_ gas operating at a temperature of 100 °C. It can be observed that the sensor responses decrease as the thickness increases. In order to determine the saturation of the current in the presence of H_2_ gas, it took almost 42 min for sensor measurements for the Z_2.5_ sample, despite it being ∼7 min for Z_0.5_, which shows the current saturation is much faster for this sensor than that for other sensors used in the current study. The highest response is 3.2 × 10^7^ for sensor Z_2.5_, followed by Z_7.5_ with a 2.2 × 10^6^ response, as seen in Figure 4e. However, as shown in Figure 4a, the maximum current value reached for Z_7.5_ is smaller than that for Z_0.5_, which shows a response of 3.3 × 10^5^. The difference comes from the conductivity change of the samples with the thickness, which leads to different *I*_air_ values. Apparently, this value is minimum for the thicker samples, which is on the order of picoamperes, while it is on the order of nanoamperes for the thinnest sample. Although Z_2.5_ displays the highest response, it should be noted that the responsivity and recovery times should also be considered. Therefore, sensor Z_0.5_, with the response and recovery times of 379 and 304 s, respectively, shows the best sensing performance at a temperature of 100 °C for 1000 ppm H_2_ gas. It should be kept in mind that the recovery current could not reach the starting current value, *I*_air_, at an operating temperature of 100 °C. The starting current value of the Z_0.5_ sensor is 2.0 × 10^–9^ A, while after recovery, it reaches a current value of 3.6 × 10^–7^ A. Although the difference is about 2 orders between the starting and recovery values, this recovery value seems negligible when it is considered that the response of the sensor is more than ∼10^5^.

The dynamic responses of four sensors are demonstrated at an operating temperature of 200 °C in Figure 4b. Sensor Z_0.5_ shows the highest response of 2.4 × 10^4^, followed by Z_2.5_ with a response of 1.8 × 10^4^ under 1000 ppm H_2_ gas conditions. In addition, the figure shows that the Z_0.5_ sensor reaches the highest current value in the presence of H_2_ gas. It is preferable for a gas sensor to reach the current value as high as possible, and the Z_0.5_ sensor is therefore even better due to the ease of detection of higher current values. Sensor Z_0.5_, due to its responsivity and recovery times of 633 and 224 s, respectively, shows the best performance among other sensors. In addition, Z_0.5_ shows the maximum response for 100 ppm at an operating temperature of 200 °C. For that reason, measurements of H_2_ concentrations under 100 ppm were performed for the Z_0.5_ sensor as shown in Figure 4f. At a temperature of 200 °C, a response of 0.84 (%84) for 10 ppm H_2_ gas is measured for Z_0.5_. Figure 4c indicates the dynamic response of sensors for 100, 500, and 1000 ppm at an operating temperature of 300 °C. Z_0.5_ demonstrates a maximum response of 7.4 × 10^4^ in comparison with other sensors. However, as shown in Figure 4b,c, the maximum current value that the Z_0.5_ sample reaches at 200 °C is about 3 times larger than that at 300 °C. Regarding the recovery point, the dynamic responses at a temperature of 300 °C show the best recovery. The sensors recover fully and reach the starting current before H_2_ is introduced into the chamber, as seen in the inset figure of semilogarithmic plots for 500 ppm H_2_ gas. Figure 4g shows the 11-cycle sensor response for sample Z_0.5_ at 300 °C for 1000 ppm H_2_ gas and the repeatability of the sensor. As seen in the figure, the response does not change significantly after 11 cycles of measurements.

Further experiments were conducted to study the hydrogen-sensing mechanism and the effect of Pd catalysis on H_2_-sensing performances of MoO_3_ nanowalls. The H_2_ gas-sensing mechanism of metal oxide-based gas sensors is generally explained as follows: oxygen molecules get adsorbed on the surface of metal oxide, which extract electrons from the conduction band of the semiconductor and reduce the conductivity of the material, resulting in the formation of negatively charged oxygen species at different operating temperatures including O_2_^–^, O^–^, and O^2–^.^62^ In the case of n-type semiconductors, this reduction in conductivity is reversed by exposure to reducing gases such as NH_3_ or H_2_. Hydrogen bonding with oxygen species that are adsorbed on the semiconductor surface removes surface-adsorbed oxygens that introduce electrons into the conduction band and lead to an increase in the conductivity of metal oxide.^42^ The formation process of negatively charged oxygen ions can be summarized as follows^63,64^
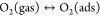

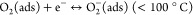

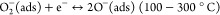

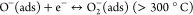


Depending on the nature and structure of materials and the behavior with different target gases, each gas sensor material has a different optimum operating temperature. The H_2_ gas-sensing mechanism of chromogenic materials such as MoO_3_ cannot be explained just by the oxygen adsorption model because H_2_ molecules and H^+^ ions not only incorporate with adsorbed oxygens but also attack different coordinated oxygens in the structure of the material in order to reduce MoO_3_ to MoO_3–*x*_. In order to improve the gas-sensing properties of metal oxide semiconductors, there have been different approaches applied so far. One of them is decorating or doping with noble metals such as Pt, Pd, or Au, which act as catalysts.^62^ To find out whether noble metals change the work function of metal oxide, there are two approaches, which are “chemical sensitization” and “electronic sensitization”.^65^ Chemical sensitization is the disassociation of the target analyte molecule called the spillover effect. In this case, the deposition of metal catalysts does not change the conductivity of the sensing material. Electronic sensitization refers to the temporary oxidation of a catalytic metal, which gives rise to the formation of a depletion layer. This depletion layer reduces the conductivity of the sensing material. Oxygens on the surface of the catalyst metal are removed when the H_2_ gas is introduced. This appears as thinning of the depletion layer, which increases material conductivity.^65^

**Figure 5:**
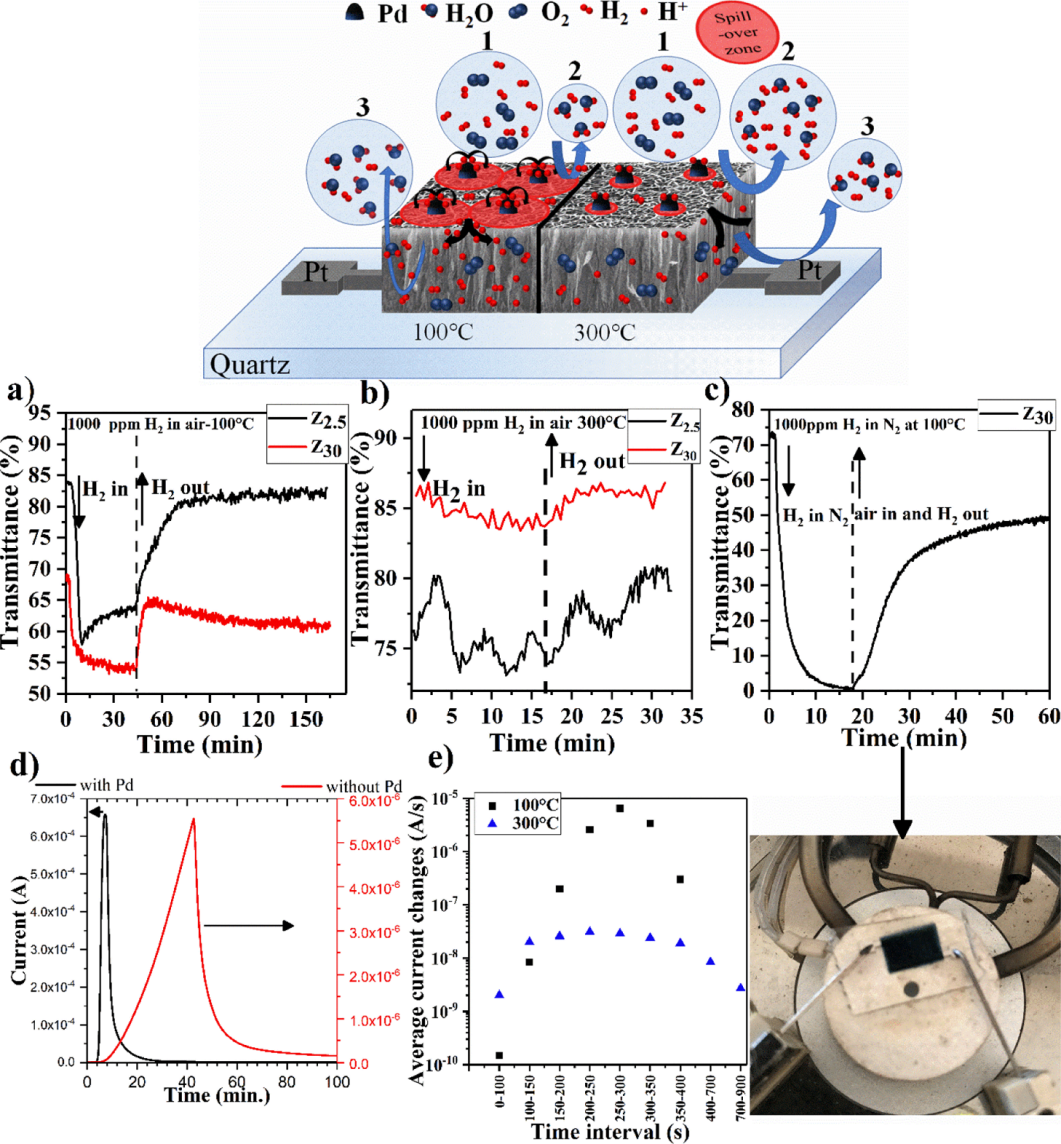
Transmittance changes of Z_2.5_ and Z_30_ for 1000 ppm H_2_ gas in air (a) at an operating temperature of 100 °C, (b) at an operating temperature of 300 °C, (c) transmittance changes of Z_30_ for 1000 ppm H_2_ gas in nitrogen at an operating temperature of 100 °C, and (d) dynamic sensor responses of Z_0.5_ with and without Pd catalysis at different operating temperatures. (e) Average current changes in sample Z_0.5_ at different operating temperatures for 1000 ppm H_2_ gas at different time intervals. The figure in the middle shows the schematic illustration of the MoO_3_ gas sensor.

Gasochromic measurements have been designed to determine the sensing mechanism related to hydrogen intercalation. The inset in Figure 5 shows the schematic illustration of the sensor materials, explaining the sensing mechanism of gas sensors produced in the study. Black dots on the surface represent the Pd nanostructures as a catalytic material. FESEM images of Pd-decorated MoO_3_ nanowalls are shown in Figure S5. The left side of the figure represents the sensing mechanism at 100 °C, while the right side shows that at 300 °C. Figure 5a,b shows the transmittance changes of samples Z_2.5_ and Z_30_ at a wavelength of 700 nm for 1000 ppm H_2_ gas at operating temperatures of 100 and 300 °C, respectively. There are no significant changes in transmittance for both sample measurements performed at 300 °C; however, significant changes of 25.5 and 15.0% transmittance are observed for samples Z_2.5_ and Z_30_, respectively, at 100 °C. These results show similarity to those of the electrical sensor measurement in which the sensor response at 100 °C is higher than that at 300 °C. Gasochromic measurements indicate that more H^+^ ions intercalate at 100 °C temperature than those at 300 °C. There might be a couple of reasons for this to observe. First of all, it might be possible with more H_2_ gas desorption from the surface at higher temperatures by removing the active number of H_2_ gas molecules, as seen in the inset figure circle number 2. For this reason, the spillover zone at 300 °C is smaller than that at 100 °C, as seen in the schematic illustration. On the other hand, the MoO3 sites reduced by the H2 gas are oxidized easily by the oxygen molecules in dry air with rising temperature^66^ because 300 °C is very close to the oxidation temperature of MoS_2_ as it is shown by the oxidation at around 380 °C from the in situ measurements. Actually, there is a trade-off between the oxidation of the sites by the oxygen in dry air and reduction by H_2_ gas. It is this trade-off that creates an equilibrium slab of active thickness, which basically determines the conductivity change of the sensors. In order to confirm this hypothesis, gasochromic measurements under H_2_ gas mixed with N_2_ were performed. As seen in Figure 5c, the transmittance change of Z_30_ under 1000 ppm H_2_ at a temperature of 100 °C and under N_2_ conditions at a wavelength of 700 nm is more than 70.0%, which is about 4.8 times higher than that of H_2_ mixed in the air. The inset figure shows the colored state of the sample. The response is much higher under N_2_ gas compared to that under dry air. It is known that N_2_ itself removes surface-absorbed oxygen species, which boosts the reduction of MoO_3_, and in the case of the nanostructure, this effect is stronger than that on compact films because more surface is exposed to N_2_ gas. This also indicates that the equilibrium slab thickness is not well enough to change the conductivity of the thick sensors compared to that of the thinner samples, indicating the higher response in the thinner samples such as Z_2.5_ and Z_0.5_.

In order to understand the effect of Pd catalysis, sensor measurements were performed for the sample Z_0.5_ without Pd loading. Figure 5d shows the dynamic sensor responses of Z_0.5_ with and without Pd catalysis at 100 °C temperature. Sensor responses obtained for Z_0.5_ under 1000 ppm H_2_ in air without Pd loading are 4074, 114, and 98 fold at 100, 200, and 300 °C operating temperatures, respectively. These values are 79.7 (at 100 °C), 207 (at 200 °C), and 754 (at 300 °C) times smaller than that obtained at the same operating temperature (see Supporting Information, Figure S4) for Pd-loaded samples. This indicates that the spillover effect occurs at different temperatures and boosts the sensor sensitivity and response time. At all operating temperatures for Pd-loaded samples, sensor responses increase significantly compared to those of bare samples. On the other hand, the sensor responsivity of Pd-loaded sample with considering temperature, shows a similar trend to that without Pd catalysis. Figure 5e shows the average current change rates of Pd-loaded Z_0.5_ sample at different operating temperatures for different time intervals of sensing times. As shown in Figure 5e, for the first 100 s of gas sensing, the average current change rate for 300 °C operating temperature is 2 nA/s, and it reaches the maximum value of 31 nA/s between 200 and 250 s. The current variation by time is almost constant through 50–400 s time intervals. However, the average current change rate starts with 150 pA/s for the first 100 s and then it reaches the maximum value of 6.5 μA/s at a time interval of 250–300 s. The rate variation is much higher than the result obtained at 300 °C. These different current change rates at different temperatures at the beginning and in the middle of the gas-sensing process can be explained by the following steps of sensing mechanism: H_2_ molecules first reach the surface, then H^+^ ions are formed by the spillover effect, these ions play a role in removing adsorbed surface oxygens, causing the conductivity change which is relatively small at the beginning of the process. Then, more H^+^ ions are formed due to the spillover effect, and the H^+^ intercalation begins in the inner sections of MoO_3_ due to the field-effect caused by the H^+^ in contact with each other at the spillover intersection zones, as seen in the schematic representation of the gas sensor in Figure 5. The reduction of MoO_3_ starts at the bulk, forming water vapor that leaves the structure, causing a dramatic increase in the current change rate as illustrated in the inset figure circle number 3 of Figure 5. Room-temperature (RT) H_2_ gas-sensing measurement for sample Z_0.5_ for 1% H_2_ gas concentration is shown in Supporting Information, Figure S6. A response of 7.5 × 10^7^ was achieved at RT. The data are included in the Supporting Information due to the long sensing and recovery times.

## 4 Conclusions

In conclusion, MoS_2_ nanowall films with four different thicknesses (40, 115, 370, and 1440 nm) deposited by RFMS were successfully turned into α-MoO_3_ by thermal oxidation without changing the morphology of the films, which are confirmed by XRD, Raman, FESEM, and XPS measurements. In situ measurements during the oxidation process were performed to control the oxidation. They also show that the nanowalls perpendicular to the substrate turn to the oxide material faster than the basal planes. It is concluded from the depth profile XPS measurement that the oxidation starts from the nanowalls found on the surface and continues through the depth of the material. The conversion from MoS_2_ to MoO_3_ is also confirmed by Raman and XRD measurements. H_2_ gas-sensing performances of MoO_3_ films were determined on films with different thicknesses at 100, 200, and 300 °C operating temperatures. The thinnest sample with a thickness of ∼40 nm shows the outstanding performance with a response of 3.3 × 10^5^-fold. Response and recovery times of 379 and 304 s, respectively, are achieved at an operating temperature of 100 °C. In addition, gasochromic measurements were performed to explain the H_2_-sensing mechanism of MoO_3_ at 100 and 300 °C. Transmittance changes of about % 25.5 and % 15.0 are achieved for samples with thicknesses of 115 and 1440 nm, respectively, at a temperature of 100 °C. Also, a further gasochromic experiment was performed for 1000 ppm H_2_ in a N_2_ atmosphere to understand the intercalation mechanism of the H^+^ ions. Elimination of the H^+^ ions by dry air is also confirmed.

## Supporting Information Available

FESEM cross-sectional images of samples Z_2.5_, Z_7.5_, Z_10_, and Z_30_ for both MoS_2_ and MoO_3_ films; FESEM images of oxide Z_7.5_ at temperatures of 400, 450, and 500 °C; TEM images of the MoO_3_ films; dynamic responses of the bare Z_0.5_ sample for 100, 500, and 1000 ppm H_2_ gas at operating temperatures of 200 and 300 °C; SEM images of Pd-decorated MoO_3_ nanowalls; and dynamic response of sensor Z_0.5_ at room temperature for 1 % H_2_ gas concentration (PDF)
