# Supplementary material for: Highly Responsive Pd-Decorated MoO3 Nanowall H2 Gas Sensors Obtained from In-Situ-Controlled Thermal Oxidation of Sputtered MoS2 Films
Source: ACS Appl Mater Interfaces. 2022 May 24;14(22):25741–52. doi: 10.1021/acsami.2c04804 (PMC9185678; doi:10.1021/acsami.2c04804)
Supplement: Supplementary file 1 — am2c04804_si_001.pdf [file am2c04804_si_001.pdf]

Supporting Information for:

Highly Responsive Pd-Decorated MoO_3 Nanowall H_2 Gas Sensors Obtained from In-Situ-Controlled Thermal Oxidation of Sputtered MoS_2 Films

Soheil Mobtakeri[a], Saman Habashyani[a], and Emre Gür[a,b]

[a]Department of Nanoscience and Nanoengineering, Graduate School of Natural and Applied Science, Atatürk University, 25240, Erzurum, Turkey
[a,b]Department of Physics, Faculty of Science, Ataturk University, 25250 Erzurum, Turkey

***Corresponding author e-mail; emregur@atauni.edu.tr**

Figure S1 has shown FESEM cross-sectional images of samples $Z_{2.5}$, $Z_{7.5}$, Z_{10}, Z_{30} for both MoS_2 and MoO_3 films. As seen from figures, nano-walls can be seen clearly from the figures. The corruption in images of oxide films is emerged during cutting of sample with glass cutter. The thickness for MoS_2 films measured are 115, 370, 474, and 1440nm for samples $Z_{2.5}$, $Z_{7.5}$, Z_{10}, and Z_{30}, respectively.

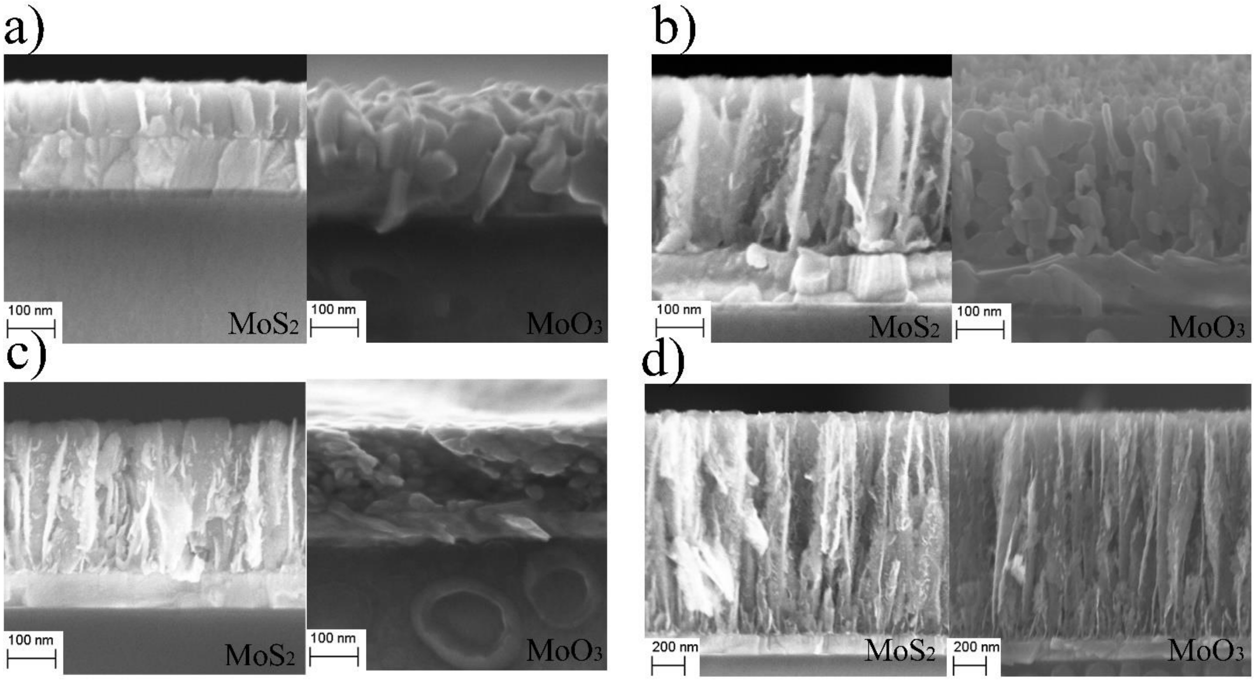

Figure S1. TEM images of MoS_2 and MoO_3 nano-walls a) $Z_{2.5}$, b) $Z_{7.5}$, c) Z_{10}, and d) Z_{30}

Figure S2 has shown FESEM images of oxide $Z_{7.5}$ at temperatures of 400, 450, and 500°C. As seen from the figures, the nano-walls becomes crispy and morphology begins to deteriorate with rising temperature structures. For this reason, the oxidation temperature of 380°C has chosen which is minimum temperature for oxidation.

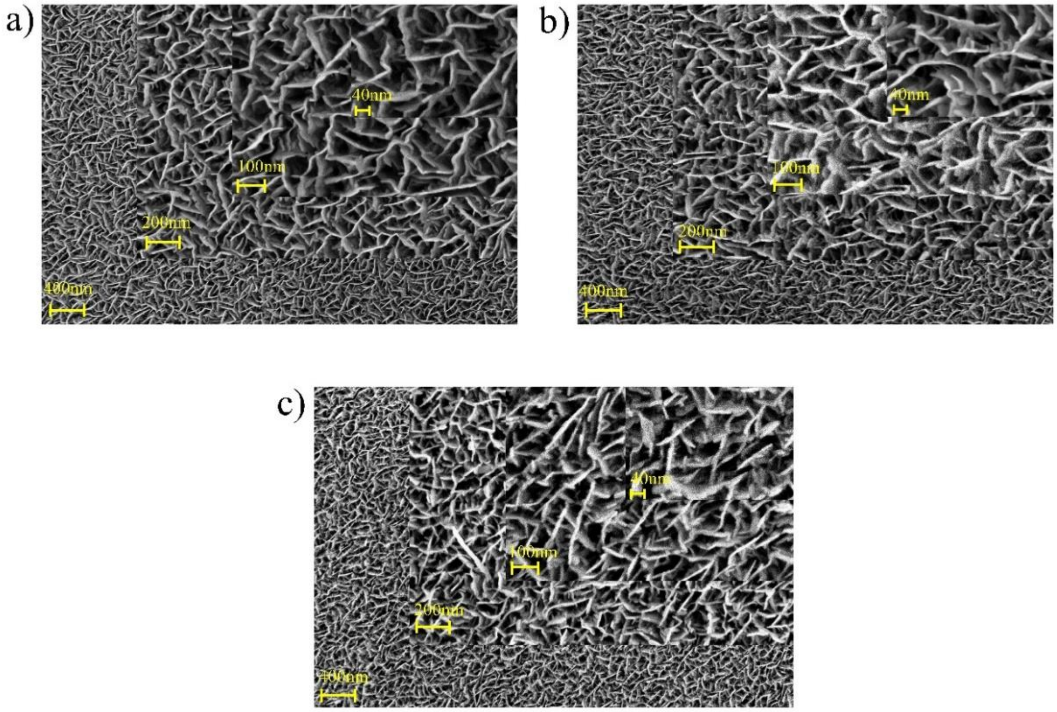

Figure S2. FESEM images of MoO_3 nano-walls obtained by thermal oxidation of MoS_2 at a) 400℃, b) 450℃, and c) 500℃.

Figure S3 has shown TEM images on the MoO_3 films which is prepared by scratching MoO_3 films from substrate on TEM grid. As seen from figure, the average distance between two MoO_3 sheet (Mo to Mo distance) is about 1.37nm which is close to theoretical distance of 1.4nm [1].

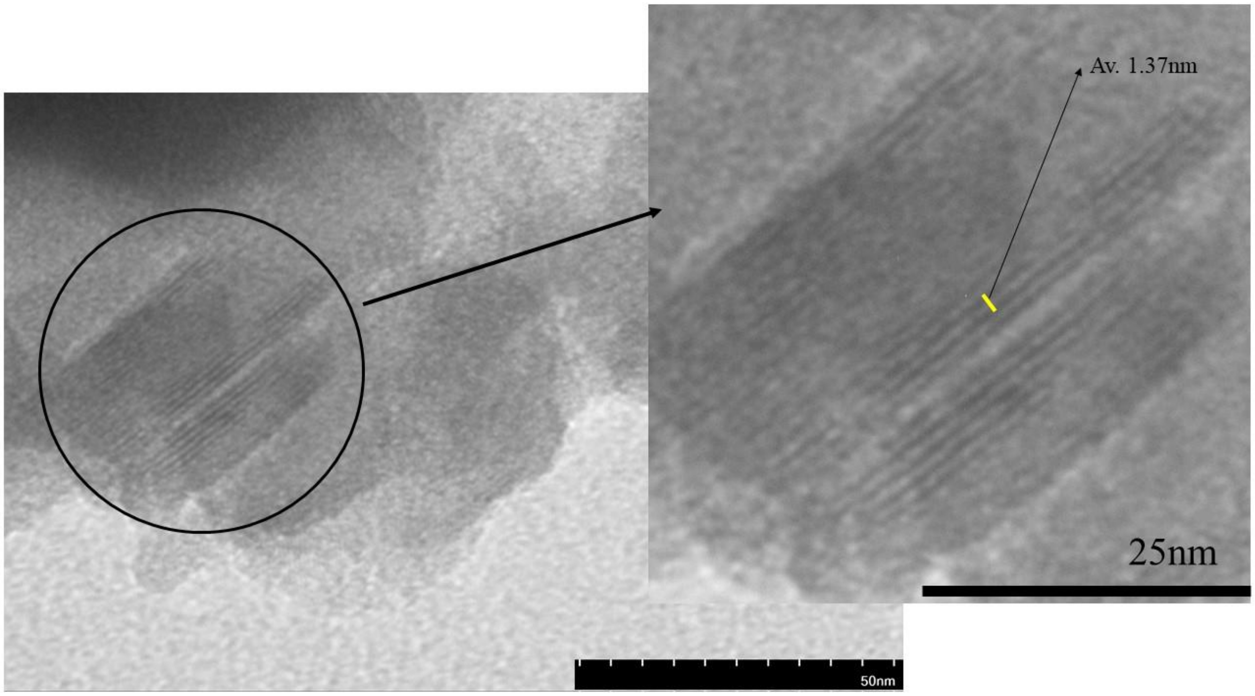

Figure S3. TEM images of MoO_3

Figure S4 has shown dynamic responses of bare $Z_{0.5}$ sample for 100, 500, and 1000ppm H_2 gas at operating temperatures of a) 200°C and b) 300°C. The response for 1000 pmm H_2 gas at 200°C is about 114 and for 300°C is about 98 times.

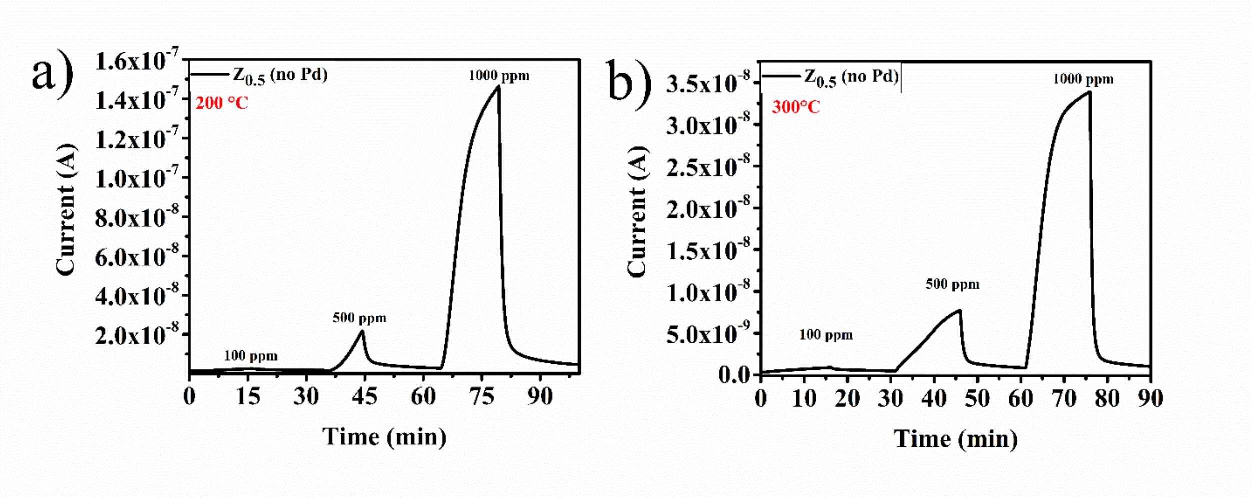

Figure S4. Dynamic responses of bare $Z_{0.5}$ at temperatures of a) 200°C and b) 300°C for 100, 500, and 1000ppm H_2 gas in air

Figure S5 has shown the SEM images of Pd decorated MoO_3 nano-walls. As seen from figure Pd nanoparticles grown on the walls after 7sec. Pd deposition with sputtering at growth pressure of 27mTorr and power of 25Watt.

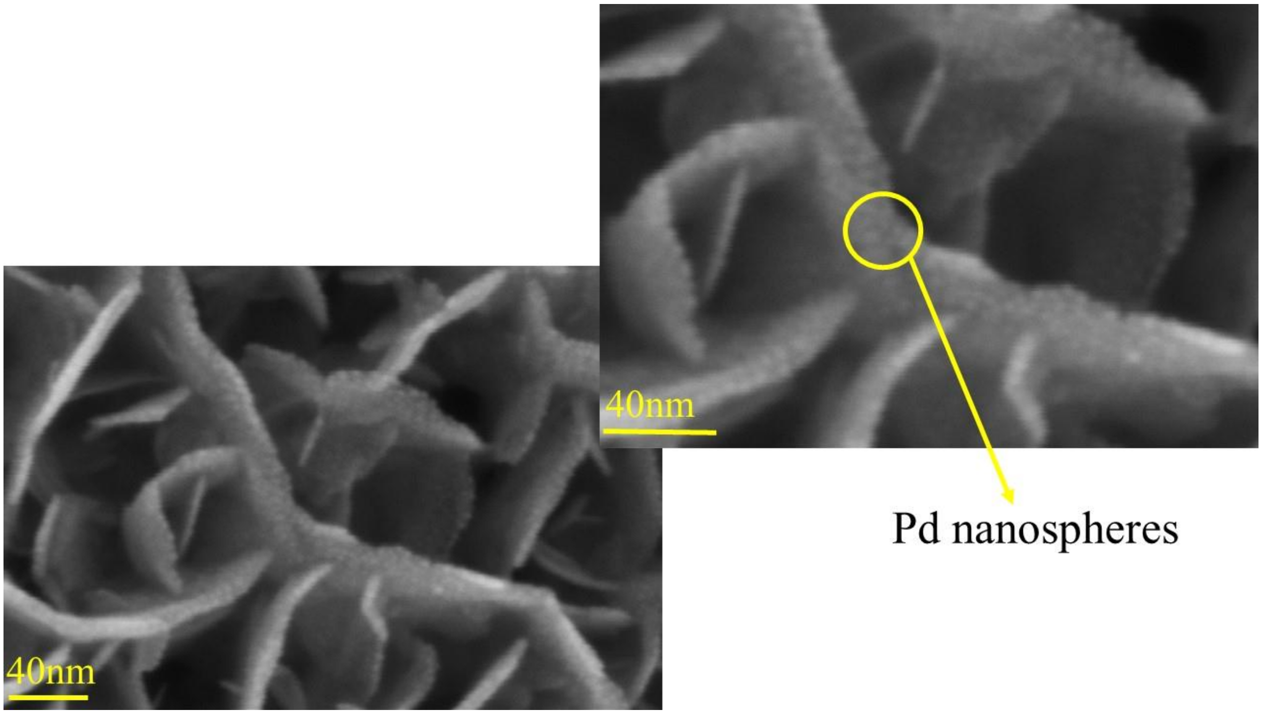

Figure S5. SEM images of MoO_3 nano-walls after deposition of Pd.

Figure S6 has shown dynamic response of sensor $Z_{0.5}$ at room temperature for %1 H_2 gas concentration. As seen from the figure, sensor is not saturated after 92 min. and current tends to rise and reaches to value of 2mA which showed the resistance of MoO_{3-x} is reached about 250Ω. After recovery for 120min. sensor just recovered %79 of its response. This indicates that to obtain full recovery sensor should be heated.

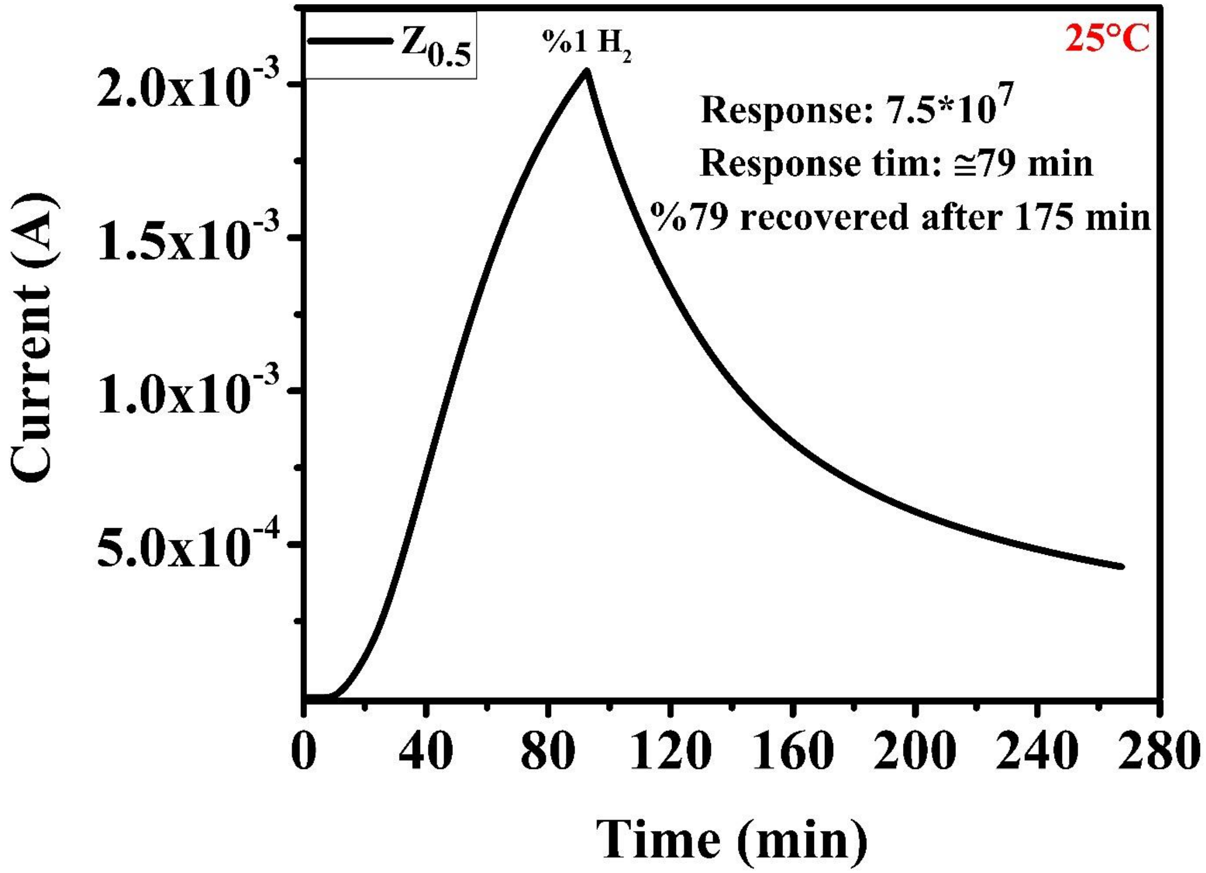

Figure S6. Dynamic response of sensor $Z_{0.5}$ at room temperature for %1 H_2 gas concentration.
